# Evaluating contributions of neuropsychological, psychiatric, and inflammatory processes to the expression of cognitive symptoms in post-acute COVID-19 syndrome

**DOI:** 10.3389/fpsyt.2025.1668380

**Published:** 2026-02-05

**Authors:** Luke S. Watson, Youssef Toubouti, Sarah M. Gray, Núria Guillén, Agnès Pérez-Millan, Lorena Rami, Raquel Sanchez-Valle, Jason K. Johannesen

**Affiliations:** 1 Sage Therapeutics, Cambridge, MA, United States; 2 Alzheimer’s Disease and Other Cognitive Disorders Unit, Service of Neurology, Hospital Clínic de Barcelona, Fundació Recerca Clínic Barcelona-IDIBAPS, & Institut de Neurociències, Faculty of Medicine and Medical Sciences, University of Barcelona, Barcelona, Spain; 3 Centro de Investigación Biomédica en Red en Enfermedades Neurodegenerativas (CIBERNED), Madrid, Spain; 4 Faculty of Computer Science, Multimedia and Telecommunications, Universitat Oberta de Catalunya, Barcelona, Spain

**Keywords:** brain-fog, neuropsychology, long COVID, rehabilitation, subjective complaints

## Abstract

**Introduction:**

Post-acute COVID-19 syndrome (PACS) has been widely associated with cognitive symptoms, however, the nature and severity of effects on cognitive function have been difficult to establish amid other aspects of PACS symptomatology. The current study used a regression modeling approach to parse unique and combined contributions of neuropsychological test performance, psychiatric symptoms, and inflammatory cytokine levels in predicting cognitive symptom severity, as measured by questionnaire responses from patient and observer perspectives.

**Methods:**

Forty-one patients presenting to a university medical center neurology clinic with cognitive complaints ≥ 4 months after COVID-19 symptom onset were included in the analysis.

**Results:**

Although pre-morbid cognitive status was estimated to be at or above-average across participants, nearly 50% performed below expectation on three or more neuropsychological tests. Subjective Cognitive Decline Questionnaire (SCD-Q) ratings were clinically elevated, both from patient (MyCog) and observer (TheirCog) perspectives, yet bivariate relationships with neuropsychological and other measures of PACS symptomatology were non-significant. When combined in regression models, 36% of variance in MyCog score was explained by measures of anxiety, premorbid intelligence, and current neuropsychological test performance. TheirCog scores were explained by a unique set of neuropsychological tests, accounting for 33% of variance cumulatively. Measures of depression, fatigue, and inflammatory cytokines concentrations did not enter either model.

**Discussion:**

Taken together, cognitive sequelae of PACS appear to be rooted in changes in brain function that are detectable by objective neuropsychological testing. Although comorbidities associated with PACS can contribute to the experience of cognitive symptoms, we find cognitive symptoms, whether self-reported or observed, to be more directly associated with neuropsychological test performance than ongoing fatigue, psychiatric symptoms, or inflammatory processes.

## 1 Introduction

The 2020 novel SARS-CoV-2 (COVID-19) pandemic left substantial healthcare challenges in its wake, the impact of which is still being felt today. Lifetime incidence rates of sustained symptomatology, known as long-COVID or post-acute COVID-19 syndrome (PACS), ranges from 7%-40% (1–3) with estimates reaching 34% in non-hospitalized patients (3). PACS is defined as the continuation or development of new symptoms beyond 4 weeks of an initial COVID-19 infection (4). The cluster of disorders can include numerous symptoms from all organ classes (4) and can be incredibly debilitating (5). Notably, alterations in central nervous system function, including cognitive complaints, fatigue, and psychiatric symptoms, are some of the most commonly reported features of PACS (3, 6).

Several provisional mechanisms have been put forth to explain central nervous system changes associated with PACS. Angiotensin-converting enzyme 2 (ACE2), an enzyme expressed in many regions of the brain (7, 8), has been identified as a primary point of cell entry for SARS-CoV-2 (9). Cellular damage from increased neuroinflammatory processes akin to those seen in neurodegenerative disease (10) and neurovascular disease (11), or from direct viral activity in the brain have also been proposed mechanisms of PACS-associated central nervous system changes. While no single or specific process has yet been established, independent lines of investigation provide preliminary evidence linking PACS symptoms to pathophysiologic changes that can profoundly impact brain function (12, 13). For example, data from the UK Biobank study compared pre- and post-SARS-CoV-2 infection changes in brain structure and discovered reductions in grey matter and total brain volume accompanied by significant impairments in performance-based tests of cognitive processing speed and executive function (14). In other work, brain alterations characterized by hypo-connectivity affecting hippocampal and fronto-cerebellar pathways and reduction in grey matter in cortical, limbic, and cerebellar structures were found to correlate with cognitive dysfunction as late as 11 months following SARS-CoV-2 infection (15). A recent study by our group found that over two-thirds of a neurology clinic sample who identified cognitive difficulty as a primary symptom exhibited reductions in executive function when tested at least 3 months post-SARS-CoV-2 infection, despite generally above-average performance on measures of premorbid cognitive status (16). Subsequent testing at 6 months reflected minimal recovery in performance-based measures, consistent with the persistence of subjective cognitive complaints.

While the term brain fog has been widely adopted colloquially in reference to a myriad of cognitive changes described by PACS patients, the scientific literature has been mixed regarding interpretations of severity, associated functional impact, and underlying etiology. Whereas some studies have reported moderate to large deficits in select domains of cognitive testing (17), others find impairment to be mild and restricted to only a subset of individuals who endorse new cognitive complaints following COVID-19 (18). Congruence between subjective symptom endorsement and impairment measured by performance-based neuropsychological tests can be difficult to establish for many reasons. For example, because 30-40% of PACS patients develop psychiatric symptoms in the months following their COVID-19 illness (6, 19), associated disturbances in sleep, energy, and volition could contribute to the experience of decline in cognitive status. Indeed, prolonged cognitive symptoms following SARS-CoV-2 infection have been described alongside psychiatric symptoms as sequelae of somatic, functional, and psychosocial consequences of the disease (20). Impressions of cognitive symptoms and their correlates may also be influenced by the manner in which key cognitive domains, such as attention, memory, and executive function, are assessed clinically, and the extent to which a patient’s experience and description of symptoms align with objective measures of cognitive test performance. Patient ratings of the Cognitive Failures Questionnaire were found to be uncorrelated with objective measures of cognitive status in one study of PACS patients, however, testing was completed using a web-based platform rather than by standard clinical assessment (21). It is also possible that subjective and objective measures of cognitive status are differentially impacted by additional aspects of the illness experience, wherein subjective appraisal may be influenced to a greater extent by common comorbidities of PACS (e.g., fatigue, psychiatric symptoms) (18) than are objective measures of neuropsychological test performance (22). With this distinction in mind, it is reasonable to question whether subjective appraisal of cognitive symptoms and tested ability should be expected to correlate, and how discrepant results between methods should be interpreted. Clarification of this issue could better prepare examiners to consider multiple sources of information in the evaluation of functional status and recovery through rehabilitation.

This study sought to better understand the clinical underpinnings of reported cognitive symptomatology in a sample of help-seeking individuals who presented to a neurology clinic post-acute COVID-19 infection with cognitive complaints. The current analysis was designed to incorporate not only an extensive battery of clinical neuropsychological tests in domains relevant to daily function, including working memory, processing speed, and executive functions, but also an estimate of premorbid cognitive status and normatively-referenced scores to aid in interpretability of test scores in the absence of a pre-COVID-19 baseline. Questionnaire-based measurement of cognitive symptoms included both subjective ratings provided by patients, as well as observer ratings provided by an informant that was used in a parallel set of analyses for comparison with patient data. Finally, appreciating that the experience and expression of cognitive symptoms may differentially relate to underlying disease processes, statistical models incorporated measures of fatigue, psychiatric symptoms, and serum markers of inflammatory activity in order to account for the impact of non-cognitive aspects of PACS on the experience of cognitive symptomatology.

## 2 Methods

### 2.1 Sample

Patients were selected from the cohort reported in Guillen and colleagues (16). Briefly, a prospective, single-center non-interventional trial was conducted to describe central nervous system symptoms in a sample reporting cognitive complaints following SARS-CoV-2 (COVID-19) infection. Inclusion and exclusion criteria were previously described (16). The present analysis was conducted in a subset of the initial sample that had data collected at study entry across various measurement domains including a cognitive symptom questionnaire, neuropsychological testing, clinical scales, and serum cytokine levels. This study was performed according to the international consensus for research with human subjects (the updated version of Helsinki’s Statement, 23) and Spanish regulations. The Hospital Clínic de Barcelona Ethics Committee (HCB/2020/1483) approved the study, and all participants provided informed consent. All data included in the current analysis were collected during the baseline assessment visit for the parent study, which occurred a mean of 10.2 months (standard deviation [SD] = 3.3, range=4-18) from the time of confirmed SARS-CoV-2 infection.

### 2.2 Assessments

#### 2.2.1 Cognitive symptoms

Participants and a designated study partner completed the MyCog and TheirCog portions of the Subjective Cognitive Decline Questionnaire (SCD-Q) (24). The SCD-Q consists of self- (MyCog) and observer-rated (TheirCog) forms to which respondents answer yes or no to questions about perceived decline in memory, language ability, and executive function. Scores of mean (M) = 12.5 (standard deviation; SD = 6.2) on MyCog and M = 11.7 (SD = 5.9) on TheirCog were obtained in an initial validation sample of individuals with mild cognitive impairment (MCI), representing significant elevation in cognitive difficulties in comparison to scores of M = 3.5 (SD = 2.8) and M = 3.2 (SD = 3.7), respectively, in healthy controls (24). Using a recommended cut-score of ≥ 7, we previously reported that 98% of MyCog and 73% of TheirCog scores fell within the pathological range among participants of the parent study (16).

#### 2.2.2 Neuropsychological test battery

Global cognitive status was assessed using the mini-mental state examination (MMSE) (25), where scores of 30–27 are considered normal, scores of 26–24 are considered borderline impairment, scores of 23–18 are considered mild impairment, and scores of 17 and below are considered at least moderate impairment (26). Pre-morbid intelligence was estimated using the Spanish Word Accentuation Test (WAT) (27). The WAT was modeled after tests of English word reading and correlates highly with full-scale measures of intellectual capacity but was designed specifically for Spanish speakers, requiring examinees to provide the correct pronunciation of 30 low-frequency Spanish words whose accents have been removed. A mean WAT total score of 24.1 (SD = 4.9) was reported for a normative standardization sample (28) with scores in the range of 17 to 27 equating to estimated full-scale IQ scores of 90-110 (29).

Language and fluency were assessed using the Boston Naming Test (BNT) (30) and Vocabulary (31), Semantic Fluency (SF) (32), and Phonemic fluency (PF) (33) tests. Learning and memory were assessed in the verbal domain using the Free and Cued Selective Reminding Test (FCSRT) (34) and in the visual domain using the Rey-Osterrieth Complex Figure Test (ROCFT - Recall) (35). Processing speed and executive functioning were measured using the Stroop Color and Word Test (SCWT) (36), Trail Making Test (TMT-A and TMT-B) (37), Symbol Search (SS), and Symbol Digits Modalities Test (SDMT) (38). Visuoconstructional processing was assessed using Copy and Time trials of the ROCFT. Working memory was assessed by Digit Span (DS) and Letter-Number Sequencing (LNS) (31). Raw scores were transformed to scalar scores adjusted for age and years of education to Spanish norms and interpreted relative to a normal mean score of 10. For the purpose of interpretation, scalar scores of 7 and below were designated as below average collectively, representing the range of possible scores falling at least 1 standard deviation below the mean, and occupied by 16% or less of the population. Scores in this range are consistent with recommendations for evaluation of mild neurocognitive disorder, according to the current Diagnostic and Statistical Manual-5 (39, 40).

#### 2.2.3 Psychiatric symptoms and fatigue

Mood and anxiety symptoms were assessed using the Beck Depression Inventory-II (BDI) (41) and Beck Anxiety Inventory (BAI) (42). Scores of 14 or more on the BDI reflect mild depression scores, and score of 16 or more on BAI reflect moderate anxiety. The Spanish version of the Multidimensional Fatigue Inventory (MFI-20) (43, 44) was used to evaluate five dimensions of fatigue: general fatigue, physical fatigue, reduced motivation, reduced activity, and mental fatigue with a total score range of 20-100. Scores of 60 or higher are considered to reflect substantial fatigue (45).

#### 2.2.4 Serum immunological panel

An immunological panel from Luminex^®^ was run on serum, measuring interferon (IFN)-α, IFN-β, and IFN-γ; interleukin (IL)-1β, IL-6, IL-8, IL-10, IL-17A, and IL-18; tumor necrosis factor (TNF)-α2; IL-1 receptor antagonist (IL-1ra); interferon-γ-inducible protein 10 (CXCL10 or IP-10); granulocyte colony-stimulating factor (G-CSF); antigen CD25; chemokine ligand 1 (CX3CL1 or fractalkine), chemokine ligand 2 (CCL2 or MCP-1), chemokine ligand 7 (CCL7 or MCP-3), and chemokine ligand 9 (CXCL9 or MIG). Samples were run according to manufacturer’s instructions. Concentrations of the following cytokines were quantified and included in the analysis: G-CSF, IL-18, IL-8, CCL2, CXCL9, and CXCL10.

### 2.3 Statistical analysis

All data were summarized using descriptive statistics. Pearson’s correlations were performed to explore bivariate relationships between SCD-Q scores and neuropsychological test scores, psychiatric symptoms, fatigue, and serum cytokine data.

Multiple linear regression analyses were performed by entering neuropsychological test scores, psychiatric symptoms, fatigue, and serum cytokine data into models as predictors of SCD-Q scores. Given the large number of independent variables and the relatively small sample available for these analyses, linear regressions were initially run to reduce the number of predictors by grouping them into families: neuropsychological tests, fatigue and psychiatric symptoms, and inflammatory markers. Each family of predictors was tested independently in models predicting MyCog and TheirCog scores using a stepwise selection approach (both forward and backward) based on the Akaike Inclusion Criterion (AIC), as implemented in the MASS package in R software (46). Variables retained from each family-specific model were then combined in an exhaustive search for the best fitting linear regression models using a branch-and-bound algorithm implemented in the Leaps package in R(Thomas Lumley based on Fortran code by 47). To minimize overfitting, the search was constrained to models with no more than five predictors, evaluating all possible combinations of up to five variables. Finally, backward selection was performed on the optimal models for MyCog and TheirCog using adjusted R^2^ to assess the proportion of variance explained by the model and to quantify the contribution of the included predictors in each case. All statistical tests were 2-sided with a significance level of 0.05. All data analyses were performed using R version 4.2.2 (48).

## 3 Results

### 3.1 Demographics

From the original 53 post-acute COVID-19 syndrome (PACS) participants characterized in the parent study (16), 41 were selected for the current analysis based on the availability of the data required for the planned analyses. The included subset was predominantly female (80%), with a mean age of 49.9 (SD = 7.9) years, mean duration of education of 14.1 (SD = 2.58, range 8-18) years, and mean time from initial onset of COVID-19 symptoms of 10.2 (SD = 3.3, range 4-18) months.

### 3.2 Cognitive symptoms

MyCog and TheirCog forms of the SCD-Q were completed for all participants. Relative to a recommended cut-off score of 7 for the upper bound of normal range, mean MyCog (M = 16.6, SD = 4.33) and TheirCog (M = 10.6, SD = 6.28) scores both indicated clinically significant elevations in cognitive difficulty. At the individual subject level, 98% of MyCog and 68% of TheirCog scores fell within the range of clinical significance. Two-tailed one-sample t-tests further indicated that both scores differed significantly from 7: MyCog (t(40) = 14.19, p < 0.001); TheirCog (t(40) = 3.81, p < 0.001). MyCog and TheirCog scores were moderately correlated (Pearson’s r = 0.41, p = 0.007).

### 3.3 Model predictors

#### 3.3.1 Neuropsychological tests

Estimates of premorbid status suggested participants were generally at or above average intellect. The mean WAT score (M = 25.5, SD = 3.41) was approximately 1 standard deviation above the WAT score associated with a full-scale IQ of 100 (cite 29). Global cognitive status measured using the MMSE was in the normal range (M = 27.8, SD = 2.44), with only 5% of MMSE scores falling into the range associated with mild cognitive impairment (< 24). Vocabulary, which tends to be preserved in the presence of decline on other measures (49), was within the average range (M = 11.4, SD = 2.62).

**Figure 1:**
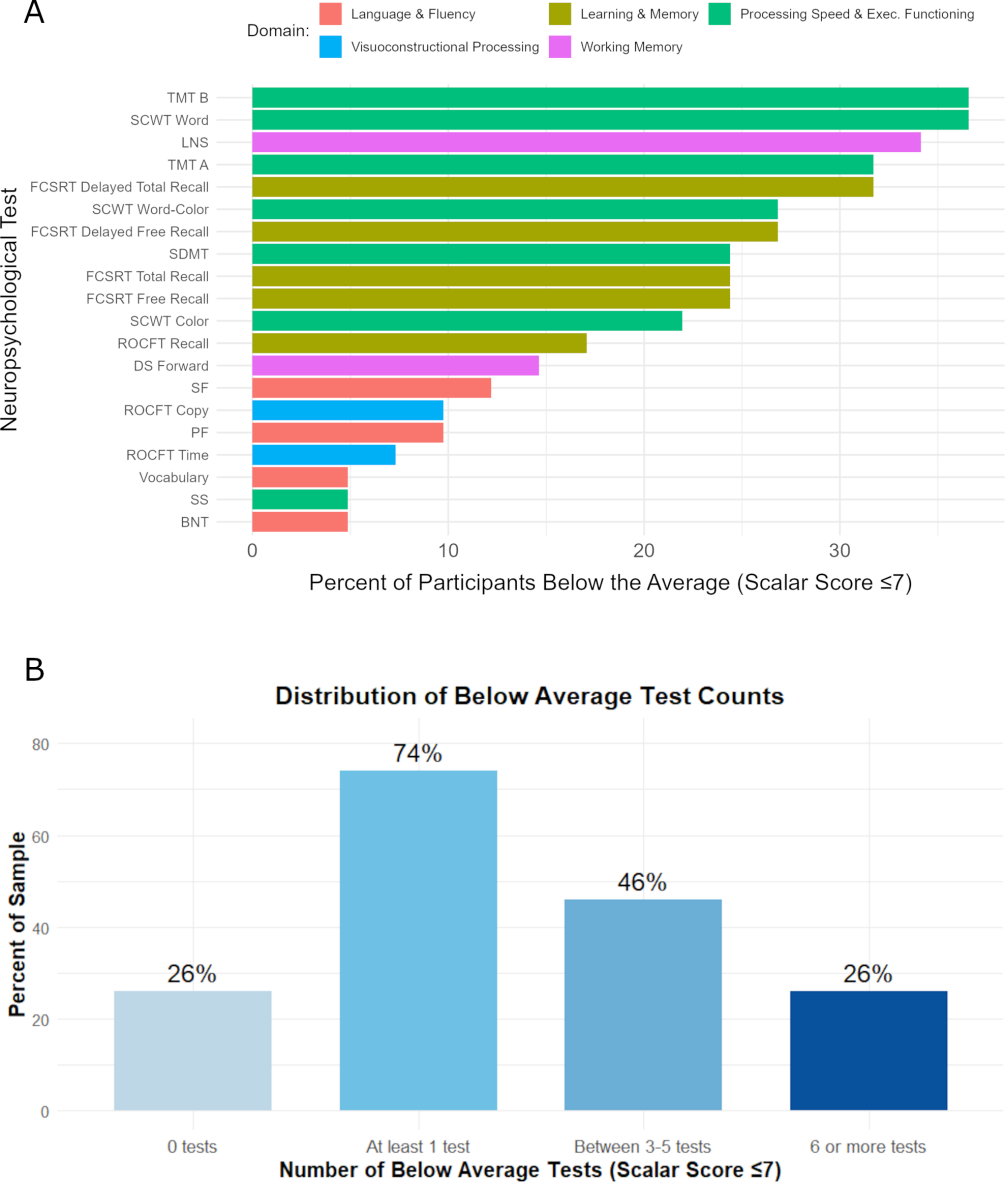
Percent of sample with below average scores on cognitive tests. **(A)** Data depict the percent of sample that have scalar scores ≤7 on each cognitive test, separated by cognitive domains. **(B)** Percent of sample that have no scores ≤7, that have at least one test score ≤7, that have at least three test scores ≤7, or that have six or more tests with score ≤7. Abbreviations: Boston Naming Test (BNT) (30), Digit Span (DS) (31), Free and Cued Selective Reminding Test (FCSRT) (34), Letter-Number Sequencing (LNS) (31), Phonemic fluency (PF) (33), Rey-Osterrieth Complex Figure Test (ROCFT) (35), Stroop Color and Word Test (SCWT) (36), Semantic Fluency (SF) (32), Symbol Digits Modalities Test (SDMT) (38), Symbol Search (SS), Trail Making Test (TMT) (37), Vocabulary (31).

Summary statistics for the full neuropsychological test battery are presented in **Table 1**. The frequency of scores at or below the normative low average range (16^th^ percentile) is reported for each measure in **Figure 1A**. Additionally, the percent of sample with no test scores reaching this threshold, and with at least 1 test, between 3–5 tests, and 6 or more tests at or below the low-average range is reported in **Figure 1B**. Most participants (>80%) had at least one test score below average, which can be expected as normal variance in a large test battery (50). Approximately 50% performed below average on 3–5 tests, and performance in this range was observed on 6 or more tests in less than 30% of participants. Accordingly, we presume that the detection rate of cognitive impairment based on neuropsychological testing would be 50% or less in this sample, depending in part on the selection of tests used in the clinical setting.

**Table 1:** Scalar score results from cognitive tests.

Test	Mean (SD)	Range
BNT	12.6 (3.35)	2 - 18
Vocabulary	11.4 (2.62)	1 - 15
SF	9.6 (2.62)	2 - 16
PF	10.1 (2.40)	5 - 15
FCSRT (Total Free Recall)	9.0 (3.32)	2 - 15
FCSRT (Total Recall)	10.0 (4.14)	2 - 18
FCSRT (Delayed Free Recall)	8.6 (3.27)	2 - 15
FCSRT (Delayed Total Recall)	10.5 (5.07)	2 - 18
ROCFT (Recall)	9.1 (1.93)	2 - 12
SCWT Word	8.4 (3.18)	2 - 18
SCWT Color	9.1 (3.01)	2 - 15
SCWT Word-Color	9.1 (2.79)	2 - 17
TMT A	9.4 (3.62)	2 - 17
TMT B	8.7 (2.91)	2 - 16
SS	10.4 (2.12)	4 - 14
SDMT	8.6 (2.66)	2 - 13
ROCFT (Copy)	12.6 (3.87)	5 - 18
ROCFT (Time)	10.5 (2.68)	2 - 16
LNS	8.7 (3.39)	2 - 16
DS	9.7 (3.28)	3 - 17

Boston Naming Test, (BNT) (30); Digit Span, (DS) (31); Free and Cued Selective Reminding Test, (FCSRT) (34); Letter-Number Sequencing, (LNS) (31); Phonemic fluency, (PF) (33); Rey-Osterrieth Complex Figure Test, (ROCFT) (35); Stroop Color and Word Test, (SCWT) (36); Semantic Fluency, (SF) (32;, standard deviation, (SD); Symbol Digits Modalities Test, (SDMT) (38); Symbol Search, (SS); Trail Making Test, (TMT) (37).

#### 3.3.2 Neuropsychiatric symptoms

A mean BAI score of 19.1 (SD = 11.10) and a mean BDI score of 14.2 (SD = 7.73) reflect moderate anxiety and mild depression in this sample, respectively. A mean MFI-20 score of 57.6 (SD = 4.98) suggests substantial endorsement of fatigue symptoms.

#### 3.3.3 Serum cytokines

Serum samples were collected at intake and measured for levels of G-CSF, IL18, IL6, CCL2, CXCL9, and CXCL10. All samples were deemed to be within normal clinical ranges, and values are described in **Supplementary Table 1**.

### 3.4 Bivariate correlation analysis

Pearson’s *r* correlations between the independent variables and both MyCog and TheirCog scores are presented separately in **Table 2**. MyCog scores were most highly correlated with anxiety (BAI) and visuospatial processing (ROCFT Time), with both relationships moderate in strength (|r| ≥ 0.40). Moderately sized (|r| ≥ 0.31) correlations were also observed between TheirCog scores and verbal memory (FCSRT Total Recall and Delayed Free Recall), visuoconstructional processing (ROCFT Time), processing speed (SS), and language abilities, as assessed by word knowledge (Vocabulary) and retrieval speed (SF).

**Table 2:** Bivariate relationships between SCD-Q ratings and neuropsychological, psychiatric, and inflammatory measures.

Test	MyCog	TheirCog
	Pearson’s *r*	Pearson’s *r*
GCSF	0.05	-0.18
IL-18	-0.07	0.03
IL-8	0.00	-0.20
CCL-2	0.06	0.12
CXCL-9	0.28 ^a^	-0.07
CXCL-10	0.11	-0.02
BAI	0.44b	0.14
BDI	0.28 ^a^	0.25
MFI	-0.16	-0.27 ^a^
WAT	-0.23	-0.26
BNT	-0.07	-0.20
Vocabulary	-0.21	-0.42 b
SF	0.05	-0.28 ^a^
PF	0.04	-0.20
FCSRT (Total Free Recall)	-0.19	-0.19
FCSRT (Total Recall)	-0.30 ^a^	-0.33 b
FCSRT (Delayed Free Recall)	-0.09	-0.37 b
FCSRT (Delayed Total Recall)	-0.22	-0.25
ROCFT (Recall)	-0.22	-0.05
SCWT Word	0.00	-0.12
SCWT Color	-0.11	-0.17
SCWT Word-Color	-0.02	-0.27 ^a^
TMT-A	-0.15	-0.21
TMT-B	-0.18	-0.01
SS	-0.28 ^a^	-0.43 b
SDMT	-0.07	-0.22
ROCFT (Copy)	-0.17	-0.08
ROCFT (Time)	-0.40 b	-0.31 b
LNS	0.14	-0.12
DS	-0.18	-0.21

a indicates a trend towards a significant relationship, p< 0.10. bindicates significance at p< 0.05.

Beck Anxiety Inventory, (BAI) (42); Beck Depression Inventory, (BDI) (41); Boston Naming Test, (BNT) (30); chemokine C-C motif ligand, (CCL); chemokine, (C-X-C motif) ligand (CXCL); Digit Span, (DS) (31); Free and Cued Selective Reminding Test, (FCSRT) (34); granulocyte colony-stimulating factor, (GCSF); interleukin, (IL); Letter-Number Sequencing, (LNS) (31); Multidimensional Fatigue Inventory, (MFI-20) (43, 44), Phonemic fluency, (PF) (33); Rey-Osterrieth Complex Figure Test, (ROCFT) (35); Stroop Color and Word Test, (SCWT) (36); Semantic Fluency, (SF) (32); Symbol Digits Modalities Test, (SDMT) (38); Symbol Search, (SS); Trail Making Test, (TMT) (37); Word Accentuation Test, (WAT) (27).

### 3.5 Predictions of subjective cognitive symptoms

The optimal model predicting MyCog score [Adjusted R^2^ = 0.363, F(4, 36) = 6.71, p < 0.001] included measures of pre-morbid intelligence (WAT), verbal memory (FCSRT - Total Recall), working memory (LNS), and anxiety (BAI): MyCog = 23.689 - 0.448*WAT - 0.348*FCSRT + 0.520*LNS + 0.169*BAI. Complete model coefficients are presented in **Table 3**.

**Table 3:** Final multiple linear regression model for MyCog.

Model Summary	R^2^	Adj. R^2^	Standard Error	p-value	Observations
	0.43	0.36	3.46	< 0.001	41
ANOVA	df	SS	MS	F	P-value
WAT	1	39.48	39.48	3.31	0.077
FCSRT (Total Recall)	1	48.73	48.73	4.08	0.051
LNS	1	94.02	94.02	7.88	0.008
BAI	1	138.03	138.03	11.56	0.002
Total	36				
	Coefficients	Standard error	T	P-value	
Intercept	23.69	4.25	5.58	0.000	
WAT	-0.45	0.18	-2.53	0.016	
FCSRT (Total Recall)	-0.35	0.14	-2.46	0.019	
LNS	0.52	0.19	2.80	0.008	
BAI	0.17	0.05	3.40	0.002	

Adjusted, (Adj.); analysis of variance, (ANOVA); Beck Anxiety Inventory, (BAI) (42); degrees of freedom (df), Free and Cued Selective Reminding Test, (FCSRT) (34); Letter-Number Sequencing, (LNS) (31); mean squares, (MS); sum of squares, (SS); Word Accentuation Test, (WAT) (27).

When retained predictors were entered in the MyCog model (**Table 4**, Model 1), the largest contribution came from neuropsychological testing (Model 2), accounting for 18% of the variance in the MyCog scores. However, psychiatric symptoms had almost equal contribution, with anxiety (Model 3) uniquely accounting for 17% variance. Separation of the neuropsychological predictors into pre-morbid (WAT) versus current cognitive function (FCSRT, LNS) suggested that pre-morbid function alone contributed minimally (Model 4, Adjusted R^2^ = 2.8%); however, it was additive when combined with measures of current function with removal of WAT reducing the explained variance to 11% (Model 5). A final exploratory model pairing anxiety with premorbid function (Model 6) explained over 21% of the variance, exceeding that accounted for by either all neuropsychological tests or anxiety alone.

**Table 4:** Variance explained by measurement domains in the final MyCog regression model.

Model	RSE	F	df	P-value	R^2^	Adj. R^2^
Model 1	3.46	6.71	4,36	<0.001	0.43	0.36
Model 2	3.92	3.96	3,37	0.015	0.24	0.18
Model 3	3.94	9.26	1,39	0.004	0.19	0.17
Model 4	4.27	2.17	1,39	0.149	0.05	0.03
Model 5	4.08	3.51	2,38	0.040	0.16	0.11
Model 6	3.84	6.48	2,38	0.004	0.25	0.22
Model 1: MyCog ~ WAT + BAI + FCSRT (Total Recall) + LNS						
Model 2: MyCog ~ WAT + FCSRT (Total Recall) + LNS						
Model 3: MyCog ~ BAI						
Model 4: MyCog ~ WAT						
Model 5: MyCog ~ FCSRT (Total Recall) + LNS						
Model 6: MyCog ~ WAT + BAI						

Adjusted, (Adj.); Beck Anxiety Inventory, (BAI) (42); degrees of freedom, (df), Free and Cued Selective Reminding Test, (FCSRT) (34); Letter-Number Sequencing,( LNS) (31); residual standard error, (RSE); Word Accentuation Test, (WAT) (27).

### 3.6 Predictions of observed cognitive symptoms

The optimal model predicting TheirCog score (Adjusted R^2^ = 0.332, F(4, 36) = 5.976, p< 0.001) consisted of neuropsychological tests of verbal memory (FCSRT Delayed Free Recall), word knowledge (Vocabulary), visuoconstructional processing (ROCFT Time), and executive function (TMT B): TheirCog = 26.910 - 0.881*FCSRT – 0.818*Vocabulary – 0.671*ROCFT + 0.885*TMT B. Complete model coefficients are presented in **Table 5**.

**Table 5:** Final multiple linear regression model for their Cog.

Model Summary	R^2^	Adj. R^2^	Standard Error	p-value	Observations
	0.33	0.33	5.14	0.001	41
ANOVA	df	SS	MS	F	P-value
FCSRT (Delayed Free Recall)	1	221.16	221.16	8.39	0.006
Vocabulary	1	179.21	179.21	6.80	0.013
ROCFT (Time)	1	53.71	53.71	2.04	0.162
TMT-B	1	176.22	176.22	6.68	0.014
Residuals	36	949.21	26.37		
Total	40				
	Coefficients	Standard error	T	P-value	
Intercept	26.91	4.47	6.02	0.000	
FCSRT (Delayed Free Recall)	-0.88	0.29	-3.02	0.005	
Vocabulary	-0.82	0.33	-2.46	0.019	
ROCFT (Time)	-0.67	0.33	-2.05	0.048	
TMT-B	0.89	0.34	2.59	0.014	

Adjusted, (Adj.); analysis of variance, (ANOVA); degrees of freedom, (df); Free and Cued Selective Reminding Test, (FCSRT) (34); mean squares, (MS); Rey-Osterrieth Complex Figure Test, (ROCFT) (35); sum of squares, (SS); Trail Making Test; (TMT) (37).

Because TheirCog ratings of cognitive symptoms related uniquely to neuropsychological tests (**Table 6**; Model 1), and no other clinical or inflammatory measure entered this model, exploratory analyses were conducted to assess the influence of anxiety and pre-morbid cognitive status for comparison with the MyCog model. Anxiety (BAI) alone was not correlated with TheirCog, explaining the observed no variance (Model 2, Adjusted R^2^ ~ 0), and had minimal influence on variance explained when force-entered with the original model predictors (Model 3). Because Vocabulary may reflect preserved abilities in the presence of cognitive decline, similar to formal measures of pre-morbid status, it was of interest to parse its influence on TheirCog in comparison to measures of current neuropsychological function included in the model (FCSRT, ROCF, TMT-B). While Vocabulary alone explained 15% of the variance (Model 4), tests of current function collectively remained strong predictors, explaining 24% of the variance (Model 5).

**Table 6:** Variance explained by measurement domains in the final their Cog regression model.

Model	RSE	F	df	p-value	R^2^	Adj. R^2^
Model 1	5.14	5.98	4,36	<0.001	0.40	0.33
Model 2	6.30	0.79	1,39	0.379	0.02	-0.01
Model 3	5.17	4.83	5,35	0.002	0.41	0.32
Model 4	5.78	8.24	1,39	0.007	0.17	0.15
Model 5	5.48	5.23	3,37	0.004	0.30	0.24
Model 1: TheirCog ~ Vocabulary + FCSRT (Delayed Free Recall) + ROCFT Time + TMT-B							
Model 2: TheirCog ~ BAI							
Model 3: TheirCog ~ Vocabulary + FCSRT (Delayed Free Recall) + ROCFT (Time) + TMT-B + BAI							
Model 4: TheirCog ~ Vocabulary							
Model 5: TheirCog ~ FCSRT (Delayed Free Recall) + ROCFT (Time) + TMT B							

Adjusted, (Adj.); Beck Anxiety Inventory, (BAI) (42); degrees of freedom, (df), Free and Cued Selective Reminding Test, (FCSRT) (34); Rey-Osterrieth Complex Figure Test, (ROCFT) (35); residual standard error, (RSE); Trail Making Test, (TMT) (37).

## 4 Discussion

This analysis was conducted in a clinical sample of patients who presented to an academic medical center’s neurology clinic with complaints of cognitive symptoms three or more months following SARS-CoV-2 infection (16). Subjective complaints were highly endorsed (98% in the clinically significant range) and, for the majority of participants, also recognized as a change from pre-COVID-19 status by an observer (68% in the clinically significant range). Cognitive complaints were accompanied by psychiatric disturbance characterized by depression, anxiety, and fatigue symptoms. However, when evaluated with a comprehensive battery of neuropsychological tests, rates of objectively-defined cognitive impairment appeared much lower, with less than 50% of participants performing neuropsychological testing in a range expected to warrant clinical attention (**Figure 1B**). A discrepancy between subjective and objectively measured cognitive symptoms appears throughout the COVID-19 literature and, at first glance, the present findings would appear consistent given the high endorsement of symptoms in the presence of generally intact impressions of neuropsychological test performance. In line with the literature, correlations between cognitive symptoms and other potential explanatory factors, including anxiety (19), depression (51, 52), fatigue symptoms (53), and inflammatory profiles, were generally modest (54, 55). Of these factors, anxiety was identified as the independent measure most highly correlated with subjectively-rated cognitive symptom severity, with a smaller but appreciable correlation also observed with depression ratings (**Table 2**). From this perspective, results are consistent with prior reports suggesting that subjective cognitive complaints of PACS are associated with aspects of psychological distress, independent of neuropsychological status (56). Nevertheless, when predictors of subjective cognitive complaints were evaluated using a multivariate modeling approach, independent and additive contributions of objective neuropsychological testing, specifically in domains of memory and pre-morbid cognitive status, also emerged. The most parsimonious model, based on anxiety and pre-morbid status, accounted for 21% of variance in MyCog score. Interpreted directionally, the combination of higher anxiety and lower pre-morbid intellectual status predicted higher severity MyCog scores. Together with these predictors, a more comprehensive model, accounting for 36% of total variance, further suggested that lower verbal memory recall in the presence of higher short-term working memory contributed to higher endorsement of cognitive complaints.

In contrast, the optimal model predicting observer-rated cognitive symptoms (TheirCog) was composed entirely of objective neuropsychological tests, involving domains of memory, visuoconstructional processing, and executive function, as well as vocabulary. Exploratory analyses of anxiety scores found no direct or interactive relationships in prediction of TheirCog. A second exploratory analysis was conducted to differentiate contributions of current neuropsychological function from estimated pre-morbid cognitive capacity by removing Vocabulary, addressing the question of whether both aspects of ability must be represented for models to capture discrepancy suggestive of decline. In this case, tests of current function explained the most variance (24%), and the contribution from Vocabulary was additive, but not required. In light of the finding that anxiety was contributory to subjective, but not observer, ratings of cognitive symptoms, input from a knowledgeable informant would appear less influenced by aspects of distress associated with symptomatology and perhaps offers a more direct appraisal of behavioral change associated with cognitive function.

Direct relationships between subjective appraisal of cognitive symptoms and objective test performance have traditionally been difficult to establish in other clinical populations. For instance, patients with persistent infection with the bacterium *Coxiella burnetiid* who report more severe cognitive complaints do not exhibit corresponding deficits on objective measures of cognitive dysfunction (57). In another example, reports of cognitive complaints in patients after a stay in the intensive care unit were found to be uncorrelated with performance on a computer-administered battery of cognitive tests (58). While some prior research has found no relationship between subjective cognitive symptoms and objective test performance in PACS (21) there are several explanations for why this relationship may vary from study to study, including the cognitive domains represented in the neuropsychological battery and individual variance in performance across the tests included. In the current study, performance on any individual neuropsychological test ranged considerably, including scores well within a range typically associated with impairment (scaled score < 3) as well as the high end of superior (scaled score ‗16) (**Table 1**). Heterogeneity in performance profiles can also be appreciated between and within the domains of neuropsychological tests (**Figure 1A**), with rates of below-average performance reaching only 35% for select measures of executive function and processing speed (TMT-B, SCWT Word Reading) and larger spread of low and intact performance noted across other cognitive domains.

The PACS participants included in the current analysis represent a healthy segment of the general population, who were screened thoroughly for pre-morbid conditions that could account for the presence of cognitive symptoms. Premorbid intelligence was estimated to be at least in the average range based on several indicators, including the Word Accentuation Test (WAT), which has been shown to correlate highly with full-scale performance measures of intellectual capacity and is resistant to mental deterioration (29), and thus useful for estimation of pre-morbid ability in a sample without measured cognitive baseline. No participant within this analysis scored below 16 on the WAT, the threshold for below average intelligence, while the mean WAT score in this sample was approximately 1 standard deviation above that associated with the midpoint of the average range of the estimated full-scale IQ equivalence (29), (**Supplementary Figure 1**). However, nearly every participant in this sample endorsed significant cognitive complaints, captured on the MyCog portion of the SCD-Q. TheirCog was also rated as elevated for most participants, with a moderately strong correlation found between these two scores; thus a basic level of consistency between sources can be assumed. When considering severity and congruence in endorsement of cognitive symptoms from patient and observer perspectives, we are inclined to interpret these data as a reliable indication that participants experienced change in cognitive status following SARS-CoV-2 infection. Although symptom validity measures were not collected in this study, the fact that this sample was recruited from a neurology clinic population would suggest that cognitive symptoms were sufficiently concerning to cause these individuals to seek evaluation and care.

The current analysis was motivated by questions regarding the extent to which subjective cognitive symptomatology in the context of PACS aligns with objective clinical indicators of impairment. Using a liberal threshold of 1 standard deviation below the population average (16^th^ percentile) to identify neuropsychological test scores that could warrant clinical attention, many participants in the current sample would likely avoid detection as impaired based on neuropsychological testing alone. While 80% of the sample was identified by below average performance on at least 1 test, only 50% met this threshold for performance on three or more tests, which extended to only 30% when the criterion for detection was set at six tests or more (**Figure 1B**). In comparison with other PACS samples described in the literature, we surmise that participants included in the current analysis are reasonably representative. In one study, performance in the range of 1–2 standard deviations below average was observed in 3 or more neuropsychological tests in approximately 60% and in 6 or more tests in approximately 40% of PACS patients (N = 49) (59), suggesting only a slightly higher rate of possible impairment in a comparably sized sample.

From this perspective, how do we reconcile the high rate of endorsed cognitive symptomatology with a neuropsychological profile that, depending in part on the selection of tests administered, could appear fundamentally intact? One possible explanation is the inherent challenge in interpreting test scores falling below expectation in the absence of a comparable baseline, from which interval changes are measured for more direct interpretation of change. The fact that high-performing individuals can remain capable of performing neuropsychological tests at or above the average range despite concerns of decline (39) has been offered elsewhere as an explanation for the observed discrepancy in correlation between subjective and objective measures of cognitive status in PACS (60). This explanation is offered as one of many alternative solutions, which may include the impact of various sources of error variance that differentially impact questionnaire- and performance-based measures as well as matters yet to be fully understood regarding the duration of PACS and stability of neuropsychological performance in appreciation of a relapsing versus remitting course of symptomatology (61). In consideration, the correlational results of the present analysis are informative in promoting an integrated model that supports a direct relationship of cognitive symptoms to objectively measured function, while also accounting for the impact of anxiety on the reporting of symptoms.

This study has several limitations, some of which are detailed in the parent study report (16). As previously described, the current analyses include a small sample size from one clinical site, and all participants were referred by healthcare providers to partake in the neuropsychological battery, which may present a referral bias. This study lacked a healthy control population due to limits imposed by the local Ethics Committee. Our results failed to replicate findings of heightened inflammatory activity, reported both in SARS-CoV-2 infection and PACS patients (62), which may be in part attributed to the limited subset of cytokines available for the current analysis. These serum cytokine measures failed to enter models based on MyCog and TheirCog; however, this outcome is consistent with a previous study that found no association between peripheral inflammation and cognitive symptoms in PACS patients (54). Notably, cytokine concentrations are only one part of the inflammatory response that may alter over time during chronic disease. Changes in neuroinflammation ascertained via PET imaging have been reported in PACS patients (55), and it is widely accepted that neuroinflammation can result in neuropsychiatric changes (63–65). Thus, it may be important to explore these relationships further in PACS patients using alternative modalities.

In summary, the current analyses led to several conclusions regarding the interpretation of subjective cognitive symptoms in PACS. First, our analyses are novel in the inclusion of both patient and observer-rated symptom scales, which were found to be correlated significantly, albeit modestly. Importantly, observer data, collected from an individual well known to the patient, lends confidence to the impression that participants experienced detectable cognitive sequelae of PACS. Using a comprehensive battery of neuropsychological tests, psychiatric scales relevant to PACS, and inflammatory cytokine measures, we next sought to identify underlying clinical features that could account for the experienced and observed cognitive symptoms. In this case, a significant portion of variance in SCD-Q scores, rated both by patient and observer, was explained by neuropsychological test performance. Given that the optimal models in each case explained roughly 35% of the variance, it is evident that our predictor set is incomplete, and substantial variance remains to be explained by factors presently unaccounted for. Nevertheless, as a second conclusion, we posit that cognitive symptoms associated with PACS are more likely attributable to changes in brain function that are detectable using routine neuropsychological testing than to epiphenomena of psychiatric symptoms, fatigue, or other COVID-19-related comorbidities. Although anxiety did enter the MyCog model, its contribution was additive to that provided by neuropsychological testing, which explained a near equal amount of variance independently. Moreover, anxiety did not appear to have any measurable influence on the relationship between objective testing and TheirCog ratings; neuropsychological testing was the only significant predictor. Remarkably, multivariate models found no influence of fatigue or inflammatory markers on cognitive symptom ratings in either the MyCog or TheirCog models, further highlighting the relative strength of the observed relationships to neuropsychological test performance. In this regard, the current results lend support to the validity of patient-reported outcome instruments, such as the SCD-Q, in the assessment of PACS-associated cognitive symptoms. Finally, although statistical analyses did uncover direct relationships between cognitive symptoms and neuropsychological test performance, suggesting that testing does capture aspects of cognitive symptomatology experienced by patients, questions remain about how valid and objective test results can be best used to inform the detection of cognitive impairment associated with PACS when differences from expected performance appear subtle or isolated to a few domains. In these cases, questionnaire-based measures, particularly those capturing input from informed observers, may be particularly valuable for identification of functional changes useful for contextualizing test results in areas suspected to be most impacted.

## Data Availability

The original contributions presented in the study are included in the article/**Supplementary Material**. Further inquiries can be directed to the corresponding author.
