# Supplementary figures and images for: Evaluating contributions of neuropsychological, psychiatric, and inflammatory processes to the expression of cognitive symptoms in post-acute COVID-19 syndrome
Source: Front Psychiatry. 2026 Feb 5;16:1668380. doi: 10.3389/fpsyt.2025.1668380 (PMC12916630; doi:10.3389/fpsyt.2025.1668380)

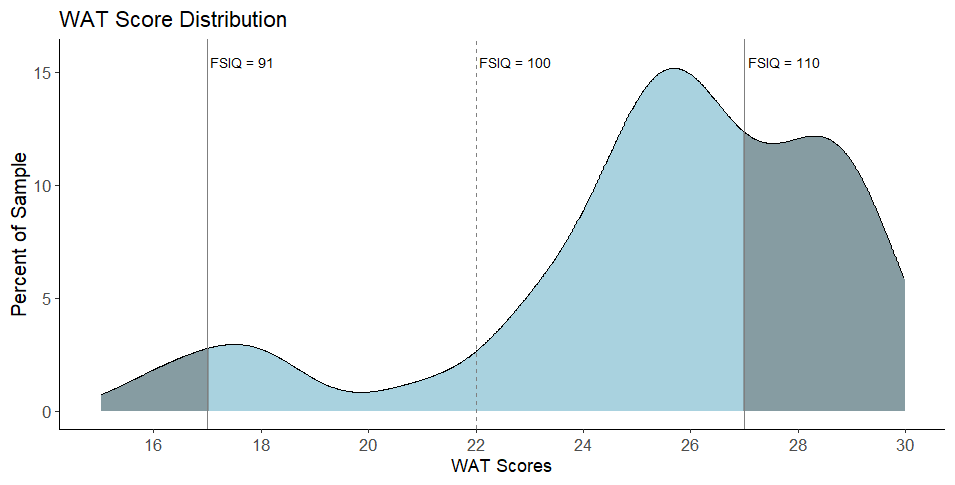

Supplement: Supplementary file 1 [file Image1.tiff]
