# Supplementary material for: Evaluating contributions of neuropsychological, psychiatric, and inflammatory processes to the expression of cognitive symptoms in post-acute COVID-19 syndrome
Source: Front Psychiatry. 2026 Feb 5;16:1668380. doi: 10.3389/fpsyt.2025.1668380 (PMC12916630; doi:10.3389/fpsyt.2025.1668380)
Supplement: Supplementary file 2 [file Supplementaryfile1.docx]

Supplementary Materials

# Supplementary Table

**Supplemental Table 1. Profile of Serum Cytokines**

|  | Mean (SD) | Range (ng/mL) |
| --- | --- | --- |
| GCSF | 36.7 (24.1) | 7.0 - 98.9 |
| IL18 | 110.0 (71.2) | 35.6 - 325 |
| IL8 | 8.11 (4.53) | 1.2 - 21.5 |
| CCL2 | 579 (169) | 252 - 1067 |
| CXCL9 | 1033 (620) | 330 - 3621 |
| CXCL10 | 72.4 (33.3) | 13.7 - 188 |

Abbreviations: Chemokine C-C motif ligand (CCL), chemokine (C-X-C motif) ligand (CXCL), interleukin (IL), (granulocyte colony-stimulating factor), nanogram per milliliter (ng/mL), standard deviation (SD).

**Supplemental Figure 1.**

Distribution of WAT scores across sample. WAT scores 17-27 represent the average range of FSIQ equivalence and are lighter shaded. Scores above 27 would indicate high-average range and below 17 would indicate low-average range, based on the FSIQ equivalence (Gomar et al. 2011), represented by darker shading on the graph. Full scale intelligence quotient (FSIQ); Spanish word accentuation test (WAT)(Del Ser et al. 1997).
